# Case of Singultis of Several Years’ Standing Cured by Acupuncture

**Published:** 1845-04

**Authors:** 


*Case of Singultis of several years' standing cured by Acupuncture,* by Dr. Emiliani.---The patient was a female, 30 years of age, of an irritable temperament, (menstruated 17 years ago.) She had suffered from singultis, with rare and short intervals, for a period of seven years, and had undergone different modes of treatment, without any beneficial result. When she placed herself under the care of the author, she appeared rather emaciated, but not particularly affected in her general health. Thus, the disease was considered of a nervous origin. The author performed acupuncture at the upper and middle part of the epigastric region, and used for this purpose from four to eight needles, which were left in the part from an hour and a half to three hours. The hiccough became less severe immediately that the needles were introduced, but returned as soon as they were removed. On the needles being re-introduced in greater number, and left there for a longer period, the hiccough was not perceived during the whole time of operation; it returned afterwards, however, but with less violence. For a month, this treatment was continued; acupuncture was repeated eight times, and the hiccough was at last so completely eradicated, that for the last three years, the patient has enjoyed complete health, and never perceived any re-appearance of her former complaint.---*Med. Times,* from *Schmidt's Jahrbuch.---Med. News.*

